# Long-Term Clinical Benefits of Pulsed Field Ablation in Paroxysmal Atrial Fibrillation: Subanalyses From the Multicenter inspIRE Trial

**DOI:** 10.1161/CIRCEP.124.013465

**Published:** 2025-04-25

**Authors:** Tom J.R. De Potter, Massimo Grimaldi, Mattias Duytschaever, Ante Anic, Johan Vijgen, Petr Neuzil, Hugo Van Herendael, Atul Verma, Allan Skanes, Daniel Scherr, Helmut Pürerfellner, Gediminas Rackauskas, Pierre Jais, Vivek Y. Reddy

**Affiliations:** Cardiovascular Center, OLV Hospital, Aalst, Belgium (T.J.R.D.P.).; Dipartimento di Cardiologia, Ospedale Generale Regionale “F. Miulli” UOC Cardiologia, Bari, Italy (M.G.).; Department of Cardiology, AZ Sint-Jan, Brugge, Belgium (M.D.).; Department for Cardiovascular Diseases, University Hospital Center Split, Croatia (A.A.).; Cardiology Department, Jessa Hospitals, Hasselt, Belgium (J.V.).; Department of Cardiology, Na Homolce Hospital, Prague, Czech Republic (P.N., V.Y.R.).; Department of Cardiology, Ziekenhuis Oost-Limburg Campus, Genk, Belgium (H.V.H.).; Division of Cardiology, McGill University Health Centre, Montreal, Quebec, Canada (A.V.).; Electrophysiology Laboratory, London Health Sciences Centre, London, Ontario, Canada (A.S.).; Division of Cardiology, Medical University Graz, Austria (D.S.).; Department für Kardiologie und Elektrophysiologie, Ordensklinikum Linz GmbH/Elisabethinen, Austria (H.P.).; Department of Cardiovascular Diseases, Centre for Cardiology and Angiology, Vilnius University, Lithuania (G.R.).; Department of Electrophysiology and Cardiac Stimulation, Centre Hospitalier Universitaire de Bordeaux (Main), Pesac, France (P.J.).; Helmsley Electrophysiology Center, Mount Sinai Fuster Heart Hospital, New York, NY (V.Y.R.).

**Keywords:** atrial fibrillation, catheters, heart atria, pulmonary veins, tachycardia

Clinical effectiveness of catheter ablation in atrial fibrillation (AF) is typically characterized by 12-month freedom from atrial tachyarrhythmia recurrence. However, freedom from AF burden may serve as a more meaningful measure of patient-centered outcomes in assessing patient quality of life (QoL), antiarrhythmic drug (AAD) use, cardioversion rates, cardiovascular hospitalizations, and repeat ablation procedures.

Pulsed field ablation (PFA) has demonstrated promising safety and effectiveness that is generally equivalent to or better than established thermal ablation technologies (eg, radiofrequency ablation and cryoablation) in treating AF. There is limited evidence for the patient-focused clinical benefits of PFA.^1^ We have previously reported the long-term safety and effectiveness of using a fully integrated biphasic PFA system with a variable loop circular catheter in combination with a multichannel PFA generator and 3-dimensional mapping system (PFA platform) in the inspIRE study (Study for Treatment of Paroxysmal Atrial Fibrillation [PAF] by Pulsed Field Ablation [PFA] System With Irreversible Electroporation [IRE]; https://www.clinicaltrials.gov; unique identifier: NCT04524364).^2,3^ Here, we report findings of a post hoc analysis of clinical benefits in inspIRE, including QoL and health care utilization outcomes.

As previously published in detail,^3^ the inspIRE study included patients aged 18 to 75 years with drug-refractory symptomatic paroxysmal AF, who underwent pulmonary vein isolation using the variable loop circular catheter integrated with the PFA platform.^3^ The inspIRE study was approved by national authorities and ethics committees, and all patients provided written informed consent. Relevant data are available upon reasonable request through the Yale Open Data Access Project (https://yoda.yale.edu).

Patient QoL was assessed pre- and post-ablation via the 20-item AF Effect on Quality-of-Life (AFEQT) questionnaire.^4^ Class I/III AAD use, repeat ablation procedure rate, direct current cardioversion incidence, and cardiovascular hospitalization rate were also assessed. Changes in the AFEQT score, proportion of patients using AADs, and proportion of patients undergoing direct current cardioversion over time were summarized descriptively. Paired *t* tests were used to compare continuous variables, and McNemar tests were used to compare nominal measures across time points. A post hoc clinical benefit success analysis was performed using a composite end point of freedom from the following postblanking: repeat ablation, cardiovascular hospitalization, new or increased AAD utilization, and direct current cardioversion. In addition, >1 repeat ablation during the blanking period was considered a failure. Predictors of clinical benefit success were explored via multivariate analysis.

Study population characteristics, and primary effectiveness and safety outcomes have been described previously.^2,3^ No primary adverse events were reported, and freedom from the primary effectiveness end point (acute pulmonary vein isolation and 12-month freedom from documented atrial arrhythmia) was 75.6%. The rate of freedom from symptomatic recurrence was 81.7%.

Of 186 evaluable patients, 176 (94.6%) completed the AFEQT questionnaires at baseline and 12 months. Compared with baseline, improvements were observed in the AFEQT composite and all subscores at 12 months (all *P*<0.001 versus baseline; Figure). The minimal clinically important difference in QoL (≥5-point improvement in AFEQT^5^) was achieved in 85.2% of patients. Patients who were free from 12-month atrial arrhythmia recurrence (asymptomatic and symptomatic) also had significantly greater improvements in AFEQT scores from baseline to 12 months versus patients with recurrence (+28.2 versus +18.8; *P*=0.0141).

**Figure.**
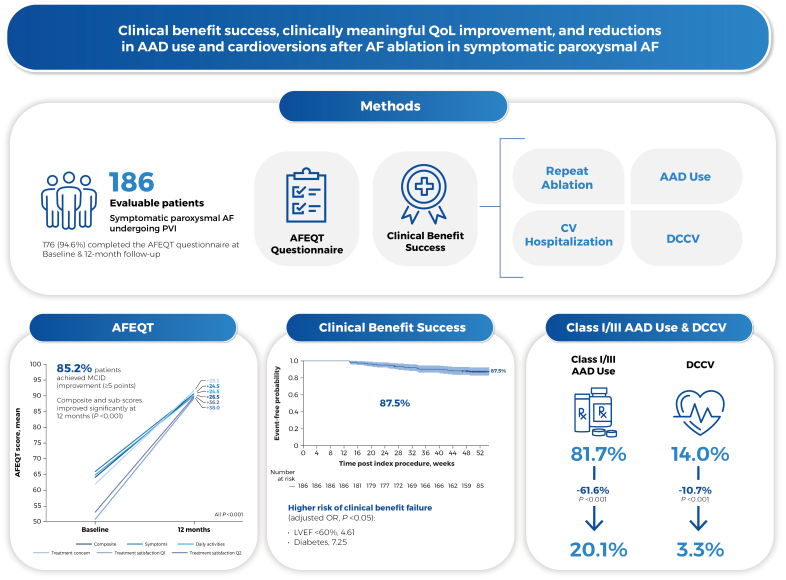
**Central illustration depicting the study design, significant improvements in Atrial Fibrillation Effect on Quality-of-Life (AFEQT) overall and subscale scores at 12 months, and Kaplan-Meier analysis of clinical benefit success in the inspIRE study.** AAD indicates antiarrhythmic drug; AF, atrial fibrillation; CV, cardiovascular; DCCV, direct current cardioversion; inspIRE, Study for Treatment of Paroxysmal Atrial Fibrillation (PAF) by Pulsed Field Ablation (PFA) System With Irreversible Electroporation (IRE); LVEF, left ventricular ejection fraction; MCID, minimal clinically important difference; OR, odds ratio; PVI, pulmonary vein isolation; and QoL, quality of life.

The proportion of patients using class I/III AADs decreased from 81.7% at baseline to 20.1% during months 6 to 12 (61.6% reduction; *P*<0.001), and the rate of direct current cardioversion decreased from 14.0% during the 12 months pre-ablation to 3.3% during the 12 months post-ablation (10.7% reduction; *P*<0.001). Six (3.2%) patients had a new class I/III AAD, while 3 (1.6%) had an increased dose of class I/III AAD postindex ablation during the evaluation period (days 91–365).

The clinical benefit success rate at 12 months was 87.5% (Figure). Multivariate logistic analysis showed that left ventricle ejection fraction <60% and diabetes were associated with higher odds of clinical benefit failure (odds ratio, 4.61 [95% CI, 1.61–13.3] and 7.25 [95% CI, 2.00–26.3], respectively; both *P*<0.05).

Patient-reported outcomes from this first-in-human trial of PFA using the variable loop circular catheter with PFA generator and 3-dimensional mapping system showed significant improvements in QoL at 12 months post-ablation, a 10.7% reduction in electrical cardioversions, and a 61.6% reduction in AAD use. The clinical benefit success rate in the current analysis was 87.5%, while, in the primary study analysis, freedom from recurrence of arrhythmia episodes of ≥30 seconds was 75.6%.^2^ Thus, by focusing only on arrhythmia monitoring, the previous analysis did not capture ≈10% of patients who had a wider scope of clinically meaningful benefit from ablation. In our post hoc multivariate analysis, left ventricle ejection fraction <60% and comorbidity of diabetes were found to be associated with a greater risk of clinical benefit failure. These findings stress the need to address existing risk factors in patients with AF, including impaired ventricular function and diabetes, to optimize outcomes of ablation therapy. The findings of this analysis highlight the potential utility of a patient-centric composite clinical benefit measure in assessing the success of catheter ablation treatment for AF and demonstrate the multifaceted benefits of PFA treatment for AF.

## Article Information

### Acknowledgments

The authors thank all inspIRE study (Study for Treatment of Paroxysmal Atrial Fibrillation [PAF] by Pulsed Field Ablation [PFA] System With Irreversible Electroporation [IRE]) personnel and patients for their valuable participation in this trial. The authors thank the following individuals for their efforts in trial execution, statistical analysis, and input during the development of this article: Christina Kaneko, Jaclyn Alcazar, Guixia Huang, Stephen Hynes, Ramona Wu, Carmen Rousseeuw, Sarah Rabau, and Nathalie Marcours. Michelle Hughes, PhD, of Lumanity Communications Inc, provided medical writing and editorial support, funded by Biosense Webster, Inc, under the direction of the authors. The inspIRE Trial Investigators: Luigi Di Biase, Jim Hansen, Sebastien Knecht, Georgios Kollias, Peter Lukac, Andrea Natale, Jan Petru, and Thomas Phlips.

### Sources of Funding

This study was funded by Biosense Webster, Inc, part of Johnson & Johnson MedTech.

### Disclosures

Dr De Potter received consulting fees and honoraria for lectures and presentations from Biosense Webster and Adagio Medical (all payments were directed to the institution). Dr Grimaldi has an unrelated patent agreement with Biosense Webster, Inc. Dr Duytschaever has served on the speakers’ bureau and as a consultant for Biosense Webster, Inc, and received research support from Biosense Webster, Inc. Dr Anic received consulting fees and has contracted research with Farapulse, Boston Scientific, Galaxy Medical, and Biosense Webster, Inc. Drs Vijgen, Neuzil, and Scherr received grant support from Biosense Webster, Inc. Dr Van Herendael received support from Biosense Webster, Inc, for congress-related activities. Dr Verma received grants from Biosense Webster, Inc, Medtronic, Bayer, and Biotronik; received consulting fees from Biosense Webster, Inc, Medtronic, Adagio Medical, Galaxy Medical, Ablacon, and Thermedical; and received honoraria for lectures from Biosense Webster, Inc, and Medtronic. Dr Skanes has served on the speakers’ bureau for Biosense Webster, Inc, and received research support from Biosense Webster, Inc. Dr Pürerfellner received consulting fees from Biosense Webster, Inc, Abbott, Boston Scientific, Biotronik, and Medtronic and received payment or honoraria for lectures or presentations from Biosense Webster, Inc, Abbott, Boston Scientific, Biotronik, and Medtronic. Dr Jais received research grants from Biosense Webster, Inc; received speaker fees from Biosense Webster, Inc; is a shareholder of Farapulse/Affera; and received speaker fees and research grants from Boston Scientific, Medtronic, and Abbott. Dr Reddy is a consultant for Biosense Webster, Inc; unrelated to this article, he serves as a consultant for and has equity in Ablacon, Acutus Medical, Affera-Medtronic, Apama Medical-Boston Scientific, Anumana, APN Health, Aquaheart, Atacor, Autonomix, Axon Therapies, Backbeat, BioSig, CardiaCare, CardioNXT/AFTx, Circa Scientific, CoRISMA, Corvia Medical, Dinova-Hangzhou DiNovA EP Technology, East End Medical, EPD-Philips, EP Frontiers, Epix Therapeutics-Medtronic, EpiEP, Eximo, Farapulse-Boston Scientific, Field Medical, Focused Therapeutics, HRT, InterShunt, Javelin, Kardium, Keystone Heart, LuxMed, Medlumics, Middlepeak, Neutrace, Nuvera-Biosense Webster, Oracle Health, Restore Medical, Sirona Medical, SoundCath, and Valcare; unrelated to this work, he has served as a consultant for Abbott, AtriAN, BioTel Heart, Biotronik, Boston Scientific, Cairdac, CardioFocus, Cardionomic, CoreMap, Fire1, Gore & Associates, Impulse Dynamics, Medtronic, Novartis, Philips, and Pulse Biosciences; and he has equity in DRS Vascular, Manual Surgical Sciences, Newpace, Nyra Medical, Surecor, and VizaraMed. Johnson & Johnson MedTech has an agreement with the Yale Open Data Access Project to serve as the independent review panel for evaluation of requests for clinical study reports and participant-level data from investigators and physicians for scientific research that will advance medical knowledge and public health. Requests for access to the study data can be submitted through the Yale Open Data Access Project site at https://yoda.yale.edu. Dr Rackauskas reports no conflicts.
